# A hybrid sampling algorithm combining synthetic minority over-sampling technique and edited nearest neighbor for missed abortion diagnosis

**DOI:** 10.1186/s12911-022-02075-2

**Published:** 2022-12-29

**Authors:** Fangyuan Yang, Kang Wang, Lisha Sun, Mengjiao Zhai, Jiejie Song, Hong Wang

**Affiliations:** 1 Department of Gynecologic Oncology, The First Affiliated Hospital of Henan Polytechnic University, Jiaozuo, 454000 Henan China; 2 Autobio Labtec Instruments Co. Ltd., Zhengzhou, 450016 Henan China

**Keywords:** Imbalanced medical data, Data sampling, Decision tree, Ensemble algorithm

## Abstract

**Background:**

Clinical diagnosis based on machine learning usually uses case samples as training samples, and uses machine learning to construct disease prediction models characterized by descriptive texts of clinical manifestations. However, the problem of sample imbalance often exists in the medical field, which leads to a decrease in classification performance of the machine learning.

**Methods:**

To solve the problem of sample imbalance in medical dataset, we propose a hybrid sampling algorithm combining synthetic minority over-sampling technique (SMOTE) and edited nearest neighbor (ENN). Firstly, the SMOTE is used to over-sampling missed abortion and diabetes datasets, so that the number of samples of the two classes is balanced. Then, ENN is used to under-sampling the over-sampled dataset to delete the "noisy sample" in the majority. Finally, Random forest is used to model and predict the sampled missed abortion and diabetes datasets to achieve an accurate clinical diagnosis.

**Results:**

Experimental results show that Random forest has the best classification performance on missed abortion and diabetes datasets after SMOTE-ENN sampled, and the MCC index is 95.6% and 90.0%, respectively. In addition, the results of pairwise comparison and multiple comparisons show that the SMOTE-ENN is significantly better than other sampling algorithms.

**Conclusion:**

Random forest has significantly improved all indexes on the missed abortion dataset after SMOTE-ENN sampled.

## Introduction

The extensive application of information technology in the medical field provides support for clinical diagnosis [1, 2]. In the process of clinical diagnosis [3, 4], the clinical decision support system (CDSS) analyzes and predicts patients' conditions according to their current disease information and the system knowledge base, so as to provide support information for diagnosis and treatment. CDSS can help doctors deal with various medical problems more efficiently and quickly with complex medical knowledge in the decision making process, so as to find more solutions for difficult and complicated diseases [5, 6].

In recent years, machine learning has been rapidly developed and widely used in clinical diagnosis [7, 8]. Clinical diagnosis based on machine learning [7] regards the disease diagnosis process as a prediction problem characterized by the clinical manifestations of the disease. According to the clinical manifestations of the disease, the feature space of the sample is established, and the existing cases and diagnostic results are used as the training set of the machine learning model, so that the new cases can be predicted.

However, the problem to be solved in a clinical diagnosis based on machine learning is sample imbalance [9, 10]. A large number of patients with some common diseases can produce a large case sample (majority sample). For rare diseases, the number of patients is very small and only a small case sample (minority sample) is produced [11, 12]. When trained on the imbalanced dataset, machine learning models tend to predict the samples into the majority [13, 14]. Although high precision can be achieved, the sensitivity of the model is extremely low, so the  model cannot correctly classify minority samples [15, 16].

At present, methods to solve the sample imbalance problem can be divided into algorithm level [17, 18] and data level [19, 20]. The algorithm level method mainly combines the characteristics of imbalanced samples to improve the algorithm appropriately to improve the sensitivity of minority. Ensemble learning [17] is a common machine learning algorithm, which outputs the results of multiple weak classifiers according to certain rules through combination training of multiple weak classifiers. SMOTE [19] is a common algorithm in the data level, which improves the sensitivity of minority by synthesizing minority samples. However, whichever method has some disadvantages, such as the ensemble algorithm does not take into account the sample distribution [21], and SMOTE is easy to synthesize “noisy sample” and “boundary sample” [22].

Based on the above description, we took the collected missed abortion [23] and diabetes [24] datasets as the research object and proposed a hybrid sampling algorithm combining SMOTE and ENN to solve the sample imbalanced problem in the clinical diagnosis. Firstly, we combing SMOTE and ENN, and used ENN to delete "noisy sample" in the majority after SMOTE synthesized the minority sample. Then, due to the understandable requirements of machine learning model for CDSS, we use the decision tree to model and predict the missed abortion dataset. Finally, the decision tree is biased to the majority in the imbalanced dataset, and we use three ensemble algorithms to ensemble the decision tree to improve the classification performance of the decision tree. The comparison experiment is divided into 3 parts: Firstly, compared with other sampling algorithms to verify the effectiveness of the proposed algorithm. Then it compared with other ensemble algorithms to achieve an accurate clinical diagnosis. Finally, statistical experiments are carried to verify whether the proposed algorithm is significantly better than the existing sampling algorithms.

The rest of this work is organized as following. Section 2 presents the medical datasets and the proposed hybrid algorithm. Section 3 is the comparative experiment and statistical experiment. Section 4 shows the discussion and analysis and Sect.  5 is conclusion.

## Datasets and methods

### Medical datasets

In this work, the missed abortion dataset collected from 2016 to 2020 is selected for research. The dataset contains 249 missed abortion samples and 112 normal samples, and contains 7 features, Age, Ethnicity, Number of Births, History of abortion, Cesarean section, Infection during Pregnancy and Thyroid test results of the pregnant women respectively. In addition, we also selected the UCI medical dataset diabetes for research. The dataset contains 500 diabetic samples and 268 normal samples, and contains 8 features, Pregnancies, Glucose, Blood Pressure, Skin Thickness, Insulin, Body mass index, Diabetes pedigree function and the Age respectively.

### Ensemble algorithm

Ensemble algorithm [17, 25], as a research hotspot in the machine learning, has been increasingly applied in clinical diagnosis. Ensemble algorithm can combine multiple weak classifiers with relatively low precision to train a strong classifier with high precision. The ensemble algorithm is generally divided into 2 stages, that is, weak classifier generation stage and weak classifier combination stage.

In the weak classifier generation stage, different generation methods are used to generate multiple weak classifiers. In the weak classifier combination stage, the multiple weak classifiers are combined by voting and the final prediction model is output. The ensemble algorithm can be divided into Bagging [26], Adaboost [27] and Random forest [28] according to different generation methods of training set and combination methods. They are introduced as following:

Bagging uses bootstrap to sample from the original training subset and obtains T training subsets with the same number of samples. T training subsets are then trained using the weak classifiers, and T weak classifiers are generated. Finally, the trained T weak classifiers are used to test the test subsets, and the prediction results are output by voting.

1 $$H_{Bagging} \left( x \right) = arg\mathop {\max }\limits_{y \in Y} \mathop \sum \limits_{t = 1}^{T} I\left( {h_{t} \left( x \right) = y} \right),y = 1,2, \cdots ,L$$

where $I\left( {} \right)$ is an indicative function, that is, $I\left( {True} \right) = 1$, $I\left( {False} \right) = 0$. $h_{t} \left( {\text{x}} \right)$ is the weak classifier, that is, $I\left( {True} \right) = 1$, $I\left( {False} \right) = 0$. In the above method, the combination order of weak classifiers $T_{1} ,T_{2} , \cdots ,T_{t}$ randomly generates $h_{t} \left( x \right)$.

Adaboost trains the weak classifier on the training subsets in turn, and the training of the subsequent weak classifier depends on the performance of the previous weak classifier. The samples with errors will appear in the training subsets of the new weak classifier with a high probability. Finally, the trained T weak classifiers are used to test the test subset, and the prediction results are output by voting.

2 $$H_{Adaboost} \left( x \right) = arg\mathop {\max }\limits_{y \in Y} \mathop \sum \limits_{t = 1}^{T} In\left( {\frac{1}{\beta }} \right)I\left( {h_{t} \left( x \right) = y} \right),y = 1,2, \cdots ,L$$

where $I\left( {} \right)$ is the indicative function, ${h}_{t}\left(x\right)$ is the weak classifier, and ${\beta }^{t}$ is the weight, which emphasizes the adjustment of sample weight and the weighting coefficient of weak classifier. Unlike Bagging, the Adaboost algorithm focuses more on samples that are prone to misclassification.

On the basis of the Bagging algorithm, Random forest uses bootstrap to sample from the original training set. Then, a number of features are selected during the training process of T weak classifiers, and these features are selected as the split points of the decision tree by comparing which features have the greatest effect on the prediction. Finally, trained T decision trees classifiers are used to test the test subsets, and the prediction results are output by voting.

3 $$D_{Random\, forest} \left( x \right) = arg\mathop {\max }\limits_{y \in Y} \mathop \sum \limits_{t = 1}^{T} I\left( {d_{t} \left( x \right) = y} \right),y = 1,2, \cdots ,L$$

where $I\left( {} \right)$ is an indicative function, $d_{t} \left( x \right)$ is the decision tree classifier。Similar to Bagging, Random forest uses weak classifiers to train T training subsets and then generates T decision tree classifiers.

### The proposed hybrid sampling algorithm

According to different sampling strategies, data sampling algorithm can be divided into over-sampling and under-sampling [29]. over-sampling algorithm improves the sensitivity of the minority by synthesizing the minority samples. SMOTE [19] is a classical over-sampling algorithm, which reduces the dataset imbalance by synthesizing new minority samples.

Suppose the minority sample is $x_{i\_min}$, and find the $k$ ($k$ is generally 5) nearest neighbor samples $x_{ik\_min}$ of $x_{i\_min}$ according to the Euclidean distance. Then the new minority sample is synthesized between the minority sample $x_{i\_min}$ and the *k*-nearest neighbor sample $x_{ik\_min}$. The synthesis formula can be given by Eq. (4).

4 $$x_{new} = x_{i\_min} + rand\left( {0,1} \right) \times \left( {x_{i\_min} - x_{ik\_min} } \right),i = 1,2, \cdots ,N$$

where rand (0,1) is a random number between 0 and 1. By setting the over-sampling rate, multiple synthesis is performed according to Eq. (4) until the two classes samples are the same.

**Fig. 1:**
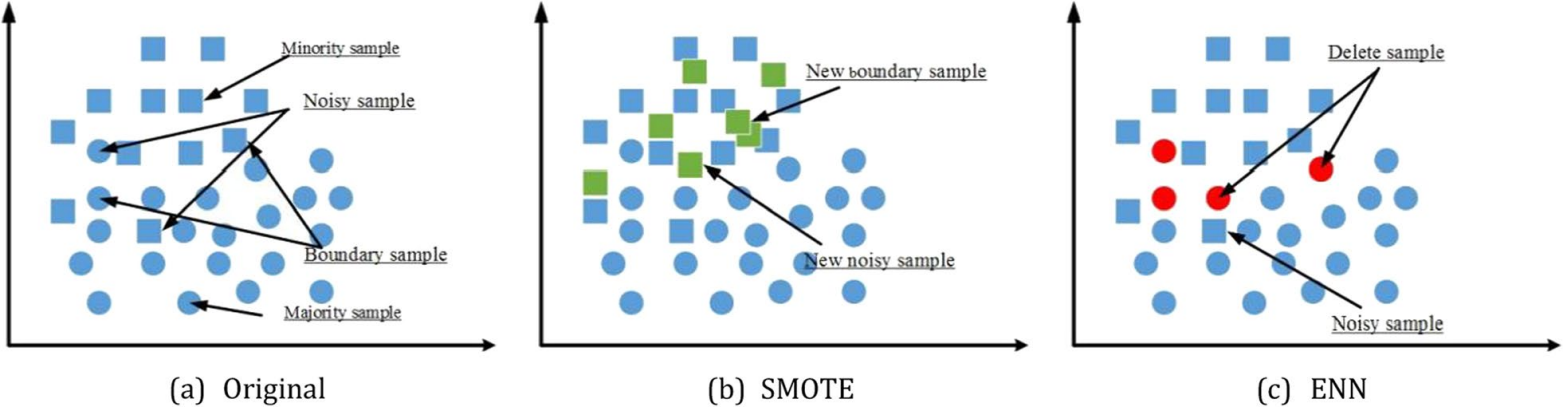
Samples simulation plot after SMOTE and ENN sampled.** a** Original dataset,** b** SMOTE dataset,** c** ENN dataset

Figure 1a shows the original dataset. Figure 1b shows that SMOTE relives sample imbalance to a certain extent, but synthetic new "noisy sample" and "boundary sample" [22, 30]. Therefore, some scholars [22, 31–33] have proposed the Borderline-SMOTE [22], Adasyn-SMOTE [31], ANS-SMOTE [32] and Gaussian-SMOTE [33] for the problems existing in SMOTE algorithm.

Recently, some scholars [21, 34] have proposed clustering over-sampling algorithms. For example, Douzas et al. [21] proposed k-means-SMOTE algorithm. The algorithm first uses k-means to cluster the dataset, then over-sampling the minority after clustering using SMOTE. Similarly, Ma et al. [34] proposed a Cure SMOTE algorithm. The algorithm uses Cure to identify and delete "noisy samples" before over-sampling again using SMOTE.

Different from the over-sampling algorithm, the under-sampling algorithm achieves the two classes balance by deleting the majority samples. ENN [20] is the common under-sampling algorithm, which deletes samples by searching whether the classes of majority samples are the same as those of the *k*-nearest neighbors. Suppose the majority samples are ${x}_{maj\_i}$, find $k$ ($k$ is generally 3) nearest neighbor samples of${x}_{maj\_i}$, and judge the class of ${x}_{maj\_i}$ and its $k$ nearest neighbor samples according to Eq. (5):

5 $$x_{j\_del} = I\left( {{\text{Class}}\left( {x_{j\_maj} - x_{jk\_maj} } \right)} \right)$$

According to Eq. (5), if the class of $x_{j\_maj}$ is different from class of the k-nearest neighbor samples, $x_{j\_maj}$ is deleted. Figure 1c shows the samples simulation plot after ENN sampled. ENN makes two classes of samples balanced by deleting “noisy sample”. However, the neighbors of majority samples are often the majority samples, and the samples that can be deleted are limited. Therefore, Tomek link [35], Instance hardness under-sampling [36], Radial based under-sampling [37] and other under-sampling algorithms have been proposed successively.

Both over-sampling and under-sampling can achieve the two classes balance, and improve the sensitivity of the minority to a certain extent. However, the specificity of the majority after sampled all declined, which may be because after sampled damaged the sample distribution of the original dataset, resulting in the decline of the specificity [38, 39].

**Fig. 2:**
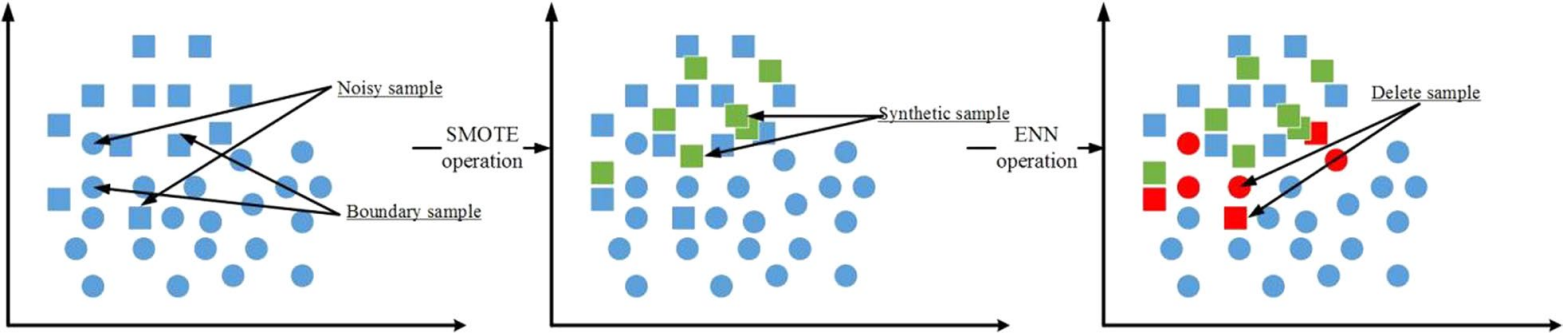
Samples simulation plot after the SMOTE-ENN sampled

In order to solve this problem, we propose a hybrid sampling algorithm combining SMOTE and ENN. The algorithm firstly uses SMOTE to over-sampling the imbalanced dataset to synthesize new minority samples. Then, ENN is used to under-sampling the oversampled dataset to delete the "noisy samples" in the minority. Figure 2 shows the samples simulation plot after the SMOTE-ENN sampled.

Observing Fig. 2, the samples simulation after the SMOTE-ENN sampled is more balanced, and the “noisy sample” synthesized by SMOTE algorithm is deleted, which is different from the dataset of SMOTE or ENN sampled alone. The steps of the SMOTE-ENN are shown in Algorithm 1.
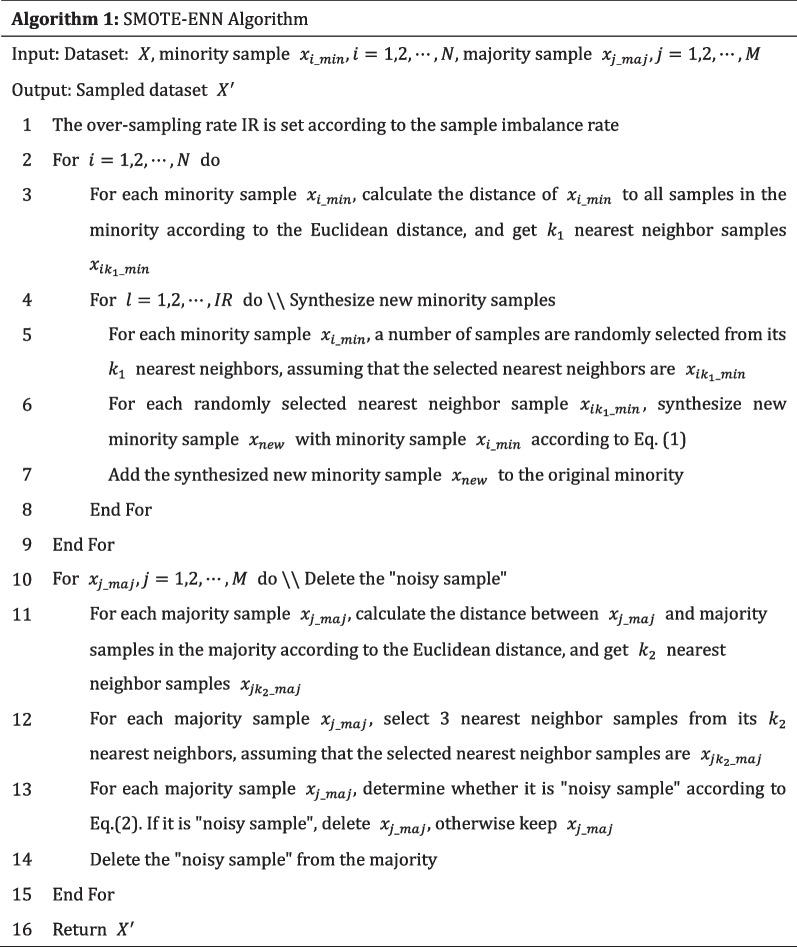


## Experimental result

### Evaluation index

Traditional evaluation indexes mainly focus on the overall classification performance, even if the minority samples are incorrectly classified, and good results will be achieved. Therefore, some scholars proposed using class classification index to evaluate its classification performance [40, 41].

If TP is used to represent the sample number of majority correctly predicted, TN to represent the sample number of minority correctly predicted, FN to represent the sample number of majority incorrectly predicted, FP to represent the sample number of minority incorrectly predicted, then:

Prediction precision of the minority (Sensitivity):

6 $$Sensitivity = TP/\left( {TP + FN} \right)$$

Prediction precision of the majority (Specificity):

7 $$Specificity = TN/\left( {FP + TN} \right)$$

Sensitivity and specificity represent the precision of minority and majority respectively. In order to reflect the classification performance of the classifier in imbalanced dataset in a more comprehensive way, this paper also gives the F-measure index for two classes, which is defined as:

8 $$F - measure = 2Recall \times Precision/\left( {Recall + Precsion} \right)$$

Recall is the same as sensitivity. Only when the recall and precision are high, the F-measure will be correspondingly high. In addition, the Matthew correlation coefficient (MCC) [42] is an evaluation index that integrates sensitivity and specificity, and is defined as:

9 $$MCC = (TP \times TN - FP \times FN)/\sqrt {\left( {TN + FN} \right)\left( {TN + FP} \right)\left( {TP + FN} \right)\left( {TP + FP} \right)}$$

When there is a large difference in the number of samples, the value of MCC is usually much smaller than sensitivity and specificity. Due to TN and FP are of the same order of magnitude, much larger than TP and FN. Therefore, MCC index can significantly reflect the influence of imbalanced datasets on the classifier, and comprehensively consider the effect of two classes.

### Experimental setting

In this section, we selected 11 traditional sampling algorithms for comparative experiments. The over-sampling algorithms are SMOTE [19], Borderline-SMOTE [22], Adasyn-SMOTE [31], Gaussian-SMOTE [33], respectively. In addition, we also select two clustering over-sampling algorithms: *k*-means-SMOTE [21] and Cure-SMOTE [34]. The under-sampling algorithms are ENN [20], Tomek link [35], Instance hardness under-sampling [36], Radial based under-sampling [37]. The hybrid algorithms are the hybrid of SMOTE and ENN [19, 20], and the hybrid of SMOTE and Tomek Link [19, 35], respectively.

In the experiments, we perform tenfold cross validation on the sampled dataset using the classification algorithm. Firstly, we use three decision trees to perform tenfold cross validation on the sampled dataset, and record the results of various indexes of the decision tree. Then, we use three ensemble algorithms to perform ensemble learning on the decision tree, and record the results of various indexes of the ensemble algorithms. Finally, we select two statistical testing methods to compare 11 over-sampling algorithms to verify the significance of SMOTE-ENN.

### The samples distribution after sampled

**Fig. 3:**
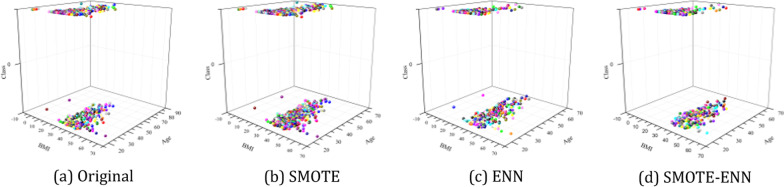
Samples scatter plot after three sampling algorithms sampled

In order to observe the samples distribution of the sampled dataset, this section presents samples scatter plot after three sampling algorithms sampled on the diabetes dataset. We plot samples scatter plot after SMOTE, ENN, and SMOTE-ENN sampled. The dataset class is selected as Z axis, and any two features are selected as X and Y axis. Figure 3 presents samples scatter plot after three sampling algorithms sampled on the diabetes dataset.

Observing Fig. 3a, it is found that the two classes of samples in the diabetes dataset differ greatly in number, and there are a large number of "noisy samples" and "boundary samples". Figure 3b–d shows samples scatter plot after SMOTE, ENN and SMOTE-ENN sampled, respectively. Observe samples scatter plot after SMOTE sampled (Fig. 3b) and find that although the number of two classes of samples is balanced, a large number of "boundary samples" are generated. In addition, there is a lot of "noisy sample" in the original diabetes dataset. By observing samples scatter plot after ENN sampled (Fig. 3c), it is found that ENN effectively deletes "noisy samples" in the minority. However, after SMOTE-ENN sampled (Fig. 3(d)), not only does SMOTE-ENN effectively synthesized the minority sample, but also deleted the "noisy sample" in the majority, thus significantly improving the sensitivity of the minority.

### Comparison with other sampling algorithms

In order to observe the sampled effect of sampling algorithms on the missed abortion and diabetes datasets, this section uses 11 sampling algorithms for comparative experiments. The 11 sampling algorithms are SMOTE (SM), Borderline-SMOTE (BSM), Adasyn-SMOTE (ASM), Gaussian-SMOTE (GSM), k-means-SMOTE (KSM), Cure-SMOTE (CSM), ENN, Tomek link (TL), Instance hardness under-sampling (IHU), Radial based under-sampling (RBU), SMOTE-Tomek link (SMTOM), and SMOTE-ENN (SMENN). In the experiment, three decision tree algorithms are used to test the sampled dataset, and the results are shown in Tables 1, 2 and 3.

**Table 1:** Results of the C4.5 on the missed abortion and diabetes dataset after sampled

Dataset	Algorithm	Original	SM	BSM	ASM	GSM	KSM	CSM	ENN	TL	IHU	RBU	SMTOM	SMENN
Missed abortion	Precision	76.0	71.0	76.0	71.2	84.2	84.7	83.7	81.8	76.0	91.2	58.2	75.2	97.1
	Sensitivity	46.4	64.1	70.7	61.9	77.9	79.5	76.7	57.1	46.4	98.2	50.0	64.7	89.8
	Specificity	90.8	82.3	80.7	79.1	89.6	89.2	89.6	94.1	90.8	82.1	66.1	83.9	100.0
	F-measure	75.5	73.0	75.6	70.3	83.7	84.3	83.1	80.5	75.5	90.1	57.8	74.1	96.9
	MCC	42.4	47.2	51.7	41.7	67.9	69.0	66.8	57.3	42.4	81.4	16.3	49.5	92.7
	AUC	70.4	74.1	74.8	73.5	86.3	86.5	86.7	77.6	70.4	90.5	59.3	74.6	91.2
	Maj/Min	249/112	249/248	249/249	249/247	249/249	249/249	249/249	219/112	249/112	112/112	112/112	249/249	114/49
Diabetes	Precision	73.5	77.9	76.8	74.9	75.0	79.8	77.8	84.6	78.5	86.1	74.8	78.3	93.0
	Sensitivity	59.7	82.4	83.6	81.2	71.8	82.0	79.8	83.2	70.9	84.0	75.4	82.6	95.0
	Specificity	81.4	73.0	68.8	67.8	78.0	77.4	75.8	85.8	83.1	88.1	74.3	73.5	90.4
	F-measure	73.6	77.7	76.1	74.4	74.9	79.7	77.8	84.5	78.5	86.0	74.8	78.0	93.0
	MCC	41.7	55.6	53.0	49.4	49.9	59.5	55.6	68.9	54.2	72.1	49.6	56.3	85.7
	AUC	75.1	79.6	76.1	78.9	79.0	84.8	80.7	85.4	80.4	86.6	74.2	80.2	93.4
	Maj/Min	500/268	500/500	500/500	500/500	500/500	500/500	500/500	240/268	445/268	268/268	268/268	476/476	229/298

**Table 2:** Results of the Randomtree on the missed abortion and diabetes dataset after sampled

Dataset	Algorithm	Original	SM	BSM	ASM	GSM	KSM	CSM	ENN	TL	IHU	RBU	SMTOM	SMENN
Missed abortion	Precision	75.0	77.2	79.1	73.6	84.3	84.4	84.4	81.8	75.0	93.2	64.8	75.8	98.8
	Sensitivity	47.3	62.5	71.5	68.4	77.5	78.3	77.9	60.7	47.3	99.1	50.9	67.1	95.9
	Specificity	89.2	88.4	85.5	78.3	90.0	89.6	90.0	92.7	89.2	85.7	76.8	83.1	100.0
	F-measure	74.9	75.0	78.4	73.3	83.7	83.9	83.9	81.7	74.9	92.4	63.2	74.9	98.8
	MCC	40.6	52.7	57.6	47.0	68.0	68.3	68.4	58.1	40.6	85.6	28.7	50.9	97.1
	AUC	72.5	79.7	81.4	79.0	85.5	84.5	85.6	80.3	72.5	92.8	57.4	79.6	98.0
Diabetes	Precision	69.1	76.5	74.1	73.1	71.5	77.4	74.6	83.9	73.4	85.1	72.4	78.1	93.2
	Sensitivity	55.2	76.6	74.6	76.6	70.8	78.2	74.0	86.2	63.8	86.9	71.3	79.4	95.4
	Specificity	76.6	76.4	73.6	69.4	71.8	76.6	75.2	81.3	79.3	83.2	73.5	76.7	90.4
	F-measure	69.1	76.5	74.1	73.0	71.3	77.4	74.6	83.8	73.4	85.1	72.4	78.0	93.2
	MCC	31.9	53.0	48.2	46.1	42.6	54.8	49.2	67.6	43.3	70.2	44.8	56.1	86.1
	AUC	65.9	76.5	74.1	73.0	71.3	77.4	74.6	83.7	71.6	85.1	72.4	78.0	92.8

**Table 3:** Results of the Reptree on the missed abortion and diabetes dataset after sampled

Dataset	Algorithm	Original	SM	BSM	ASM	GSM	KSM	CSM	ENN	TL	IHU	RBU	SMTOM	SMENN
Missed abortion	Precision	75.4	74.3	73.0	69.2	81.8	82.1	82.6	78.7	75.4	90.0	58.9	71.0	97.0
	Sensitivity	44.6	65.3	66.7	61.1	75.1	75.1	75.9	44.6	44.6	80.4	60.7	64.3	91.8
	Specificity	90.8	81.9	78.7	76.3	87.6	88.0	88.4	95.0	90.8	97.3	57.1	77.1	99.1
	F-measure	74.8	73.5	72.6	68.6	81.3	81.4	82.1	75.8	74.8	88.8	58.9	70.6	96.9
	MCC	40.8	47.9	45.7	37.9	63.1	63.6	64.8	48.4	40.8	78.8	17.9	41.7	92.7
	AUC	73.9	74.2	74.4	74.6	85.6	85.2	84.6	77.1	73.9	90.0	59.2	74.6	92.8
Diabetes	Precision	74.8	77.0	76.5	75.0	75.2	79.1	79.1	84.0	76.0	88.5	73.7	79.4	91.9
	Sensitivity	58.2	76.2	83.4	79.6	73.4	78.2	79.6	82.1	63.1	84.0	74.3	81.3	94.3
	Specificity	84.6	77.8	68.4	70.0	77.0	80.0	78.6	85.8	84.3	92.5	73.1	77.3	88.6
	F-measure	74.9	77.0	75.8	74.7	75.2	79.1	79.1	83.9	76.0	88.2	73.7	79.3	91.8
	MCC	44.4	54.0	52.4	49.8	50.4	58.2	58.2	67.8	48.6	76.8	47.4	58.7	83.4
	AUC	76.7	81.3	77.8	79.7	79.4	86.1	84.8	90.1	80.1	91.7	76.6	81.5	94.5

As shown in Tables 1, 2 and 3, the sensitivity and specificity other indexes of the three decision tree algorithms on the missed abortion and diabetes datasets are all poor. This shows that the sample imbalance greatly damages the classification performance of the decision tree algorithms. In clinical diagnosis, this result is obviously unacceptable. In the over-sampling algorithms, the sensitivity indexes of the decision tree algorithms on the sampled dataset have been significantly improved. Among them, decision tree algorithms have the best classification performance on the missed abortion dataset after *k*-means-SMOTE sampled. Similarly, *k*-means-SMOTE has the best sampled effect on the diabetes dataset, and the MCC indexes of the three decision tree algorithms are 59.5%, 54.8% and 58.2%, respectively, which is significantly better than other over-sampling algorithms. In addition, the sampled effect of the Cure-SMOTE is also better than other over-sampling algorithms. This shows that the clustering over-sampling algorithm significantly better than the over-sampling algorithm.

In the under-sampling algorithms, decision tree algorithms have the best classification performance on the missed abortion and diabetes datasets after IHU sampled. But overall, ENN and IHU are better than the over-sampling algorithm, while Tomek link and RBU are worse. The specificity index of C4.5 decreases significantly after RBU sampled, which may be due to the blind deletion of some important majority samples by RBU. In the hybrid sampling algorithms, SMOTE-ENN has the best sampled effect on the missed abortion dataset, and all indexes are better than SMOTE-Tomek link. Compared with the original dataset, the imbalance rate of the dataset is improved after sampled. Among them, the Maj/Min index of the diabetes dataset after SMOTE and k-means-SMOTE sampled all reached 500/500. In addition, SMOTE-ENN has the best sampled effect in all the sampling algorithms, mainly because SMOTE-ENN not only synthesized the minority samples, but also deleted the “noisy samples” in the majority. More importantly, Randomtree is also the best classification performance in the decision tree algorithms.

### Comparative experiments of ensemble algorithms

Clinical diagnosis based on machine learning has extremely high requirements for diagnostic results. Thus three ensemble algorithms are proposed to ensemble decision tree. Similarly, we select 11 sampling algorithms to sample the missed abortion and diabetes dataset, and use Random forest, Adaboost and Bagging to test the sampled dataset. Among them, the weak classifier for Adaboost and Bagging is Randmotree. Results of ensemble algorithms on the missed abortion and diabetes datasets after sampled are shown in Tables 4, 5 and 6.

**Table 4:** Results of Random forest on the missed abortion and diabetes datasets after sampled

	Algorithm	Original	SM	BSM	ASM	GSM	KSM	CSM	ENN	TL	IHU	RBU	SMTOM	SMENN
Missed abortion	Precision	76.0	76.9	78.0	73.5	84.3	84.0	83.8	81.8	76.0	93.0	63.4	75.5	98.2
	Sensitivity	50.0	64.9	72.3	69.6	79.1	79.1	79.1	60.7	50.0	86.6	53.6	68.7	93.9
	Specificity	89.2	86.3	83.1	77.1	88.8	88.4	88.0	92.7	89.2	98.2	72.3	81.5	100.0
	F-measure	75.9	75.4	77.6	73.3	83.9	83.7	83.5	81.1	75.9	92.4	62.6	75.0	98.1
	MCC	43.0	52.5	55.8	46.9	68.2	67.8	67.3	58.1	43.0	85.4	26.4	50.4	95.4
	AUC	74.5	81.6	82.2	80.0	86.9	86.0	87.2	82.6	74.5	93.8	57.9	81.2	99.9
Diabetes	Precision	74.8	81.7	82.7	82.1	77.6	81.9	81.5	87.8	79.5	90.4	78.0	84.1	95.1
	Sensitivity	61.6	85.8	88.2	87.0	77.6	81.4	82.8	87.7	69.8	87.7	78.0	87.0	97.0
	Specificity	82.4	77.2	76.2	76.4	77.6	82.4	80.2	87.9	85.6	92.9	78.0	80.9	92.6
	F-measure	75.0	81.5	82.1	81.6	77.6	81.9	81.5	87.8	79.5	90.3	78.0	83.9	95.1
	MCC	44.6	63.2	64.9	63.8	55.2	63.8	63.0	75.5	56.2	80.7	56.0	68.0	90.0
	AUC	81.9	89.5	89.0	89.2	86.4	90.5	89.7	95.0	85.9	96.2	85.5	91.0	98.9

**Table 5:** Results of Adaboost on the missed abortion and diabetes datasets after sampled

	Algorithm	Original	SM	BSM	ASM	GSM	KSM	CSM	ENN	TL	IHU	RBU	SMTOM	SMENN
Missed abortion	Precision	76.0	77.1	78.5	74.7	83.7	84.3	83.9	82.1	78.6	93.0	65.6	75.9	98.2
	Sensitivity	50.0	66.1	72.3	70.9	79.9	79.1	80.3	61.6	58.0	86.6	52.7	69.5	93.9
	Specificity	89.2	85.9	83.9	78.3	87.1	88.8	87.1	92.7	88.8	98.2	76.8	81.5	100.0
	F-measure	75.9	75.8	78.0	74.6	83.5	83.9	83.7	81.5	78.6	92.4	64.2	75.4	98.1
	MCC	43.0	53.1	56.6	49.3	67.2	68.2	67.6	58.8	49.5	85.4	30.4	51.4	95.6
	AUC	74.5	80.9	81.9	80.1	85.9	85.2	85.9	79.9	73.5	92.9	58.6	80.7	96.9
Diabetes	Precision	69.2	75.4	73.3	72.3	72.0	76.7	74.1	81.9	74.5	84.0	71.4	78.8	92.2
	Sensitivity	55.6	78.2	72.6	73.0	71.2	78.6	74.6	82.1	68.3	85.4	67.9	77.9	94.6
	Specificity	76.6	72.4	74.0	71.6	72.8	74.8	73.6	81.7	77.8	82.5	74.6	79.6	89.1
	F-measure	69.2	75.3	73.3	72.3	72.0	76.7	74.1	81.9	74.3	84.0	71.2	78.8	92.2
	MCC	32.3	50.7	46.6	44.6	44.0	53.4	48.2	63.7	45.6	67.9	42.6	57.6	84.1
	AUC	66.1	75.3	73.3	72.3	72.0	76.7	74.1	81.9	73.0	84.0	71.3	78.8	91.9

**Table 6:** Results of Bagging on the missed abortion and diabetes dataset after sampled

	Algorithm	Original	SM	BSM	ASM	GSM	KSM	CSM	ENN	TL	IHU	RBU	SMTOM	SMENN
Missed abortion	Precision	75.7	76.0	78.6	72.0	84.6	83.7	84.5	82.1	75.7	91.9	58.1	76.7	97.6
	Sensitivity	48.2	66.9	71.9	66.0	78.3	78.3	78.7	61.6	48.2	86.6	53.6	69.9	91.8
	Specificity	89.6	83.5	84.3	77.5	90.0	88.4	89.6	92.7	89.6	96.4	62.5	82.7	100.0
	F-measure	75.5	75.1	78.0	71.7	84.1	83.3	84.1	81.5	75.5	91.5	58.0	76.2	97.5
	MCC	42.1	51.2	56.7	43.8	68.7	67.0	68.7	58.8	42.1	83.4	16.1	53.1	94.2
	AUC	73.8	81.7	82.1	79.3	86.9	86.2	87.1	82.5	73.8	93.0	59.5	80.8	98.9
Diabetes	Precision	74.1	81.0	81.0	79.0	75.6	81.4	79.9	86.4	79.8	87.7	76.7	81.6	95.1
	Sensitivity	63.1	86.2	88.2	86.4	78.2	84.8	83.6	86.6	75.4	87.7	78.7	87.0	97.7
	Specificity	80.0	75.0	72.2	70.0	72.8	77.6	75.8	86.3	82.0	87.7	74.6	75.4	91.7
	F-measure	74.1	80.5	80.1	78.1	75.5	81.2	79.7	86.4	79.6	87.7	76.7	81.1	95.0
	MCC	43.0	61.6	61.2	57.2	51.1	62.6	59.6	72.8	56.9	75.4	53.4	62.8	90.0
	AUC	78.7	88.3	86.3	86.7	83.4	89.4	87.4	93.4	84.5	94.7	82.9	88.5	98.5

As shown in Tables 4, 5 and 6 that the classification performance of the three ensemble algorithms on the original missed abortion and diabetes datasets is very poor, each index is only slightly higher than the classification performance when using decision tree alone. The classification performance of the three ensemble algorithms on the sampled dataset has been improved significantly. In the over-sampling algorithms, Gaussian-SMOTE has the best sampled effect on the missed abortion dataset, and the MCC indexes of Random forest, Bagging and Adaboost algorithms are 86.9%, 85.9% and 86.9% respectively. Similarly, *k*-means-SMOTE has the best sampled effect on the diabetes dataset. In the under-sampling algorithms, IHU has the best sampled effect on the diabetes dataset, and the MCC indexes of Random forest, Bagging and Adaboost algorithms are 80.7%, 67.9% and 75.4%, respectively. In addition, the sampled effect of ENN on the diabetes dataset is also better than that of the over-sampling algorithm.

In the hybrid sampling algorithms, SMOTE-ENN has a better sampled effect on the missed abortion and diabetes datasets, and the indexes are significantly better than SMOTE-Tomek link. In addition, the indexes of the three ensemble algorithms on the missed abortion dataset after SMOTE-Tomek link sampled are lower than those of Gaussian-SMOTE and IHU. Observing the three ensemble algorithms shows, Random forest has the best classification performance on the sampled missed abortion dataset, especially in SMOTE-ENN after sampled the sensitivity and MCC indexes are 93.9% and 95.4% respectively, which are consistent with the previous experimental results. Similarly, Random forest has the same result on the diabetes dataset after SMOTE-ENN sampled, and the sensitivity and MCC indexes are 97.0% and 90.0% respectively. In summary, we select SMOTE-ENN as the sampling algorithm for the dataset and Random forest as the diagnosis algorithm, which is the best combination and has the best classification performance.

### Statistical test

In order to further compare the results of different sampling algorithms and observe whether there are significant differences between algorithms, statistical test is required for the experimental results. We used two statistical tests, pairwise comparison and multiple comparisons, respectively.

In pairwise comparison, Wilcoxon test [43] is selected to compare all sampling algorithms. The Wilcoxon test can be described as following:

By calculating the difference in the results in the two sampling algorithms on different indexes, and ranking according to the absolute value of the difference starting from 1. If two identical values exist, the average of the ordinal number is used as the ranked value for both.

The sign is added to the ranked values according to the positive and negative differences, and the positive ranked values are added together to obtain $R+$, and the negative ranked values are added together to obtain $R-$. The minimum value of the two is selected as the T value.

Find the threshold value according to the significance level, and the null hypothesis is that there is no difference between the algorithms. If the $T$ value is less than or equal to the threshold value, the null hypothesis can be rejected and a significant difference between the algorithms can be considered.

According to the principle of the Wilcoxon test, we select the results of 6 indexes as the data values in the experiment, the significance level is $\alpha =0.05$ and the null hypothesis is that all algorithms have the same result. The Wilcoxon test based on Random forest, Adaboost and Bagging is shown in Table 7.

**Table 7:** Wilcoxon test based on Random forest, Adaboost and Bagging

Algorithm	Original	SM	BSM	ASM	GSM	KSM	CSM	ENN	TL	IHU	RBU	SMTOM
Random forest												
R +	21	21	21	21	21	21	21	21	21	21	21	21
R-	0	0	0	0	0	0	0	0	0	0	0	0
Hypothesis	Rejected	Rejected	Rejected	Rejected	Rejected	Rejected	Rejected	Rejected	Rejected	Rejected	Rejected	Rejected
Selected	SMENN	SMENN	SMENN	SMENN	SMENN	SMENN	SMENN	SMENN	SMENN	SMENN	SMENN	SMENN
Adaboost												
R +	21	21	21	21	21	21	21	21	21	21	21	21
R-	0	0	0	0	0	0	0	0	0	0	0	0
Hypothesis	Rejected	Rejected	Rejected	Rejected	Rejected	Rejected	Rejected	Rejected	Rejected	Rejected	Rejected	Rejected
Selected	SMENN	SMENN	SMENN	SMENN	SMENN	SMENN	SMENN	SMENN	SMENN	SMENN	SMENN	SMENN
Bagging												
R +	21	21	21	21	21	21	21	21	21	21	21	21
R-	0	0	0	0	0	0	0	0	0	0	0	0
Hypothesis	Rejected	Rejected	Rejected	Rejected	Rejected	Rejected	Rejected	Rejected	Rejected	Rejected	Rejected	Rejected
Selected	SMENN	SMENN	SMENN	SMENN	SMENN	SMENN	SMENN	SMENN	SMENN	SMENN	SMENN	SMENN

Due to the results of 6 groups indexes, when significance level $\alpha =0.05$, the critical value is 2, that is, the maximum value for rejecting the null hypothesis is 2. From the results that during the test of the sampled missed abortion dataset using Random forest, Adabbost and Bagging, the null hypothesis can be rejected, that is, SMOTE-ENN is significantly better than other sampling algorithms.

In the multiple comparisons, we use the Friedman test to compare all sampling algorithms. For each index, algorithms to rank by the result in descending order. If the results are the same, use the average of the ranked values as the respective ranked values. For each algorithm, the average value ${R}_{j}^{2}$ is obtained as the comparison value, using Friedman test:

10 $$\chi_{F}^{2} = \frac{12N}{{k\left( {k + 1} \right)}}\left[ {\mathop \sum \limits_{j = 1}^{k} R_{j}^{2} - \frac{{k\left( {k + 1} \right)^{2} }}{4}} \right]$$

where $N$ is the number of indexes, $k$ is the number of algorithms, and $R_{j}$ is the average value of each algorithm. To obtain better statistical results, $\chi_{F}^{2}$ distribution is transformed into $F_{F}$ distribution, and get:

11 $$F_{F} = \frac{{\left( {N - 1} \right)\chi_{F}^{2} }}{{N\left( {k - 1} \right) - \chi_{F}^{2} }}$$

The $F_{F}$ distribution has $k - 1$ and $\left( {k - 1} \right)\left( {N - 1} \right)$ degrees of Friedman. Then, experimental results of Random forest, Adaboost and Bagging are compared respectively, and the significance level $\alpha =0.05$ is adopted, where the null hypothesis is that there is no difference between the 12 sampling algorithms. According to the Eqs. (10) and (11), when $N = 6$, Friedman test result is:

$$\chi_{F}^{2} = \frac{12 \times 6}{{12 \times 13}}\left[ {8.33^{2} + 7.17^{2} + 10.00^{2} + 3.67^{2} + 4.50^{2} + 4.83^{2} + 6.17^{2} + 9.17^{2} + 2.00^{2} + 11.83^{2} + 9.33^{2}+ 1.00^{2}- \frac{{2028 }}{4}} \right] = 57.69$$

$$F_{F} = \frac{{\left( {6 - 1} \right) \times 57.69}}{{6\left( {12-1} \right) - 57.69}} = 34.71$$

When $${\upalpha } = 0.05$$, $${\text{F}}\left( {12,60} \right) = 1.9$$$$17$$, that since $$34.71\gg 1.917$$, the null hypothesis can be rejected, and the 12 sampling algorithms are considered to have significant differences. Similarly, Friedman test results obtained from Adaboost and Bagging experimental results are 30.45 and 49.28 respectively, which are also much larger than 1.917, so the null hypothesis is rejected.

## Discussion and analysis

In all sampling algorithms, the classification performance of decision tree on missed abortion and diabetes datasets after 4 over-sampling algorithms sampled is significantly better than that of the Tomek link and ENN. The sampled effect of IHU is significantly better than the over-sampling algorithm, and the MCC indexes of Randomtree on missed abortion and diabetes datasets are 85.6% and 70.2%, respectively. The SMOTE-ENN has the best sampled effect on the missed abortion dataset, and the average values of precision, sensitivity, specificity, F-measure, MCC and ACU of Randomtree are 98.8%, 95.9%, 100.0%,98.8%, 97.1% and 98.0%, respectively, which is significantly better than SMOTE-Tomek link. Similarly, precision, sensitivity, specificity, F-measure, MCC and AUC indexes of Randomtree on the diabetes dataset after SMOTE-ENN sampled are 93.2%, 95.4%, 90.4%, 93.2%, 86.1% and 92.8%, respectively. This shows that the SMOTE-ENN not only synthesizes the minority samples, but also deletes the "noisy samples" in the majority.

In addition, by observing the samples scatter plot of the diabetes dataset after sampled, it is found that Maj/Min after deletion by the ENN not reach 112/112, while Maj/Min after synthesis by the SMOTE algorithm reaches 249/248. Therefore, the "noisy sample" can be deleted by the ENN is limited. Unfortunately, due to the working principle of the SMOTE, the synthesized samples partially fall in the majority. Therefore, it is necessary to deletion the samples after SMOTE synthesis, the main purpose of which is to delete the "noisy sample" blindly synthesized by SMOTE. SMOTE-ENN firstly uses SMOTE to synthesize the minority samples, and then uses ENN to delete the "noisy sample" in the majority. Although the Maj/Min of the diabetes dataset after SMOTE-ENN sampled is only 114/49, all the indexes of the three decision trees are optimal.

In Experiment, Randomtree has the best classification performance in the three decision tree algorithms. Therefore, we use ensemble algorithm to ensemble Randomtree. Comparing the three ensemble algorithms, Random forest, Bagging and Adaboost all have poor classification performance on the not sampled missed abortion and diabetes datasets, especially the sensitivity index. Similarly, the sampled effect of the over-sampling algorithm is better than the under-sampling algorithm. The sampled effect of IHU is significantly better than other over-sampling algorithms, and MCC indexes of Random forest, Adaboost and Bagging on the diabetes dataset are 80.7%, 85.4%and 67.9%, respectively. Overall, ensemble algorithms have the best classification performance on the missed abortion and diabetes datasets after SMOTE-ENN sampled. This shows that the ensemble algorithms have the same results on the missed abortion and diabetes datasets. In addition, through the ensemble of Adaboost and Bagging on Randomtree, it is found that the classification performance has been significantly improved after the ensemble.

In order to further test the validity of the SMOTE-ENN, the pairwise comparison and multiple comparisons are used to statistically test the 12 sampling algorithms, respectively. In pairwise comparison, precision, sensitivity, specificity, F-measure, MCC and AUC indexes of the three ensemble algorithms on the sampled missed abortion dataset are taken as values. When the significance level is 0.05($\mathrm{\alpha }=0.05$), pairwise tests based on Wilcoxon are rejecting the null hypothesis. This means that the SMOTE-ENN has significant advantages than other sample algorithms. Similarly, in the multiple comparisons, precision, sensitivity, specificity, F-measure, MCC and AUC indexes of the three ensemble algorithms on the sampled missed abortion dataset are also taken as values. When the significance level is 0.05($\mathrm{\alpha }=0.05$), no matter which ensemble algorithm is used for the test, the SMOTE-ENN is significantly better than other sampling algorithms.

In general, the high sample imbalance seriously damages the classification performance of ensemble algorithm. Sampling algorithms can solve the influence of sample imbalance to a certain extent after sampled the missed abortion and diabetes datasets. Overall, the over-sampling algorithm is better than the under-sampling algorithm. However, IHU has the best sampled effect in the single sampling algorithms. The sampled effect of SMOTE-Tomek is worse than that of some single sampling algorithms. The sampled effect of the SMOTE-ENN is optimal, which is mainly because it not only synthesized the minority samples, but also deleted the "noisy samples" in majority. In addition, Random forest has the best classification performance in the ensemble algorithms. Therefore, Random forest is used as the diagnosis algorithm for the missed abortion and diabetes datasets.

## Conclusion

Medical datasets are often imbalanced, and different diseases have different sample numbers. Some diseases have only a small number or even one case sample, which greatly increases the diagnostic effectiveness of machine learning algorithms. In clinical diagnosis, minority samples are also extremely important, and the prediction of difficult diseases can greatly help doctors to treat patients in advance. A hybrid sampling algorithm combining SMOTE and ENN is proposed to study the missed abortion diagnosis. Firstly, SMOTE is used to synthesize the minority samples so that there is a balance between the majority and the minority. Then, ENN is then used to under-sampling the synthesized dataset to delete the "noisy samples" in the majority. Finally, the ensemble algorithm is used to model and predict the synthesized dataset. Randomtree has the best classification performance on missed abortion and diabetes datasets after SMOTE-ENN sampled, and all indexes are significantly better than other sampling algorithms. In addition, Random forest has the best classification performance in all the ensemble algorithms. Therefore, Random forest is selected as the diagnosis algorithm for the missed abortion and diabetes datasets.

## Data Availability

The use of data in this study is limited, and the data set can be obtained from the corresponding author (Hong Wang) according to reasonable requirements.
